# Hyperoside Attenuate Inflammation in HT22 Cells via Upregulating SIRT1 to Activities Wnt/*β*-Catenin and Sonic Hedgehog Pathways

**DOI:** 10.1155/2021/8706400

**Published:** 2021-06-10

**Authors:** Jin Huang, Liang Zhou, Jilin Chen, Tingbao Chen, Bo Lei, Niandong Zheng, Xiaoqiang Wan, Jianguo Xu, Tinghua Wang

**Affiliations:** ^1^Institute of Neuroscience, Basic Medical College, Kunming Medical University, Kunming, Yunnan 650500, China; ^2^Department of Neurology, Zhenxiong County People Hospital, Zhaotong, Yunnan Province, China; ^3^Department of Animal Zoology, Kunming Medical University, Kunming, Yunnan Province, China; ^4^Department of Neurosurgery, West-China Hospital, Sichuan University, Chengdu, Sichuan 610041, China; ^5^Neurosurgery of the People's Hospital of Leshan, Leshan 614000, China

## Abstract

Neuroinflammation plays important roles in the pathogenesis and progression of altered neurodevelopment, sensorineural hearing loss, and certain neurodegenerative diseases. Hyperoside (quercetin-3-O-*β*-D-galactoside) is an active compound isolated from Hypericum plants. In this study, we investigate the protective effect of hyperoside on neuroinflammation and its possible molecular mechanism. Lipopolysaccharide (LPS) and hyperoside were used to treat HT22 cells. The cell viability was measured by MTT assay. The cell apoptosis rate was measured by flow cytometry assay. The mRNA expression levels of interleukin-1*β* (IL-1*β*), interleukin-6 (IL-6), interleukin-8 (IL-8), and tumor necrosis factor-*α* (TNF-*α*) were determined by quantitative reverse transcription polymerase chain reaction. The levels of oxidative stress indices superoxide dismutase (SOD), reactive oxygen species (ROS), catalase (CAT), glutathione (GSH), and malondialdehyde (MDA) were measured by the kits. The expression of neurotrophic factor and the relationship among hyperoside, silent mating type information regulation 2 homolog-1 (SIRT1) and Wnt/*β*-catenin, and sonic hedgehog was examined by western blotting. In the LPS-induced HT22 cells, hyperoside promotes cell survival; alleviates the level of IL-1*β*, IL-6, IL-8, TNF-*α*, ROS, MDA, Bax, and caspase-3; and increases the expression of CAT, SOD, GSH, Bcl-2, BDNF, TrkB, and NGF. In addition, hyperoside upregulated the expression of SIRT1. Further mechanistic investigation showed that hyperoside alleviated LPS-induced inflammation, oxidative stress, and apoptosis by upregulating SIRT1 to activate Wnt/*β*-catenin and sonic hedgehog pathways. Taken together, our data suggested that hyperoside acts as a protector in neuroinflammation.

## 1. Introduction

Neuroinflammation is a chronic inflammation of brain tissue, which plays an important role in the pathogenesis and progression of altered neurodevelopment, sensorineural hearing loss, and certain neurodegenerative diseases [1–5]. In the early stages of the central auditory pathway, noise-induced hearing loss and conductive hearing loss are related to neuroinflammation [6]. In addition, neuroinflammation contributes to neuronal death and neurological deterioration by increasing the production of proinflammatory factors and oxidative stress [7]. Numerous studies have shown that hippocampal neurons are susceptible to neuroinflammatory and cause neurological complications [8]. However, there are still no effective agents or methods to restore and prevent neuronal damage caused by neuroinflammation. Thus, the identification of effective inflammatory protective candidate agents is crucial.

Hyperoside (quercetin3-O-*β*-D-galactoside) is an active compound isolated from Hypericum plants. It has antioxidant and anti-inflammatory activities, decreasing calcium overload and inhibiting apoptosis [9, 10]. Previous studies have confirmed that hyperoside effectively prevents neurological complications caused by neuroinflammation. In the hyperglycemia-induced oxidative stress and inflammation acute diabetes model, the administration of hyperoside prevented cognitive dysfunction, neuroinflammation, and oxidative stress caused by DM through the TNF-*α*/NF-*κ*B/caspase-3 signaling pathway [11]. In diseases such as Parkinson's disease, hyperoside acts as a protective agent by attenuating LPS-induced activation of microglia [12]. So far, there are few studies on the protective effect of hyperoside on neuroinflammation, and the mechanism has not been fully elucidated.

Lipopolysaccharide (LPS) is widely used to activate the innate immune system. Previous studies have shown that LPS is usually used to prepare neuroinflammation models induced by inflammatory response [13, 14]. In this study, we exposed HT22 cells to LPS to mimic a cellular model of neuroinflammation. Simultaneously, the protective effect of hyperoside on neuroinflammation and its possible molecular mechanism were studied through this model. We found that hyperoside protects HT22 cells from LPS-induced inflammation; oxidative stress and apoptosis are closely related to SIRT1 levels. Further analysis showed that hyperoside alleviated LPS-induced inflammation, oxidative stress, and apoptosis by upregulating SIRT1 to activate Wnt/*β*-catenin and sonic hedgehog pathways.

## 2. Materials and Methods

### 2.1. Reagents and Drugs

LPS, hyperoside, and 3-(4,5-dimethyl-2-thiazolyl)-2,5-diphenyltetrazolium bromide (MTT) were purchased from Sigma-Aldrich (St. Louis, MO, USA). Dulbecco's modified Eagle medium containing 10% fetal bovine serum (FBS) was purchased from Gibco (Carlsbad, USA). 100 U/ml penicillin and 100 mg/ml streptomycin were purchased from Sigma-Aldrich (St. Louis, MO, USA). LiCl and sonic hedgehog agonist SAg were purchased from Merck (San Diego, CA, USA). Primary antibodies against Bcl-2, Bax, caspase-3, BDNF, NGF, SIRT1, Wnt1, *β*-catenin, Shh, Patch, and GAPDH and secondary antibodies were all purchased from Cell Signaling (Boston, MA, USA).

### 2.2. Cell Culture and Treatments

The HT22 murine neuronal cell line was purchased from Kunming Cell Bank of the Chinese Academy of Sciences (Kunming, China). HT22 cells were seeded at 2 × 10^4^ cells/well in a 6-well plate and cultured in the DMEM with 10% fetal bovine serum, 100 U/ml penicillin, and 100 *μ*g/ml streptomycin at 37°C in a 5% CO_2_ humidified incubator. When HT22 reached 70% confluency after 24 h, cell transfection pretreated with different concentrations of hyperoside and LPS (1 *μ*g/ml) was performed.

### 2.3. Cell Viability

Cell viability was determined by MTT assay. Briefly, the HT22 cells seeded into 24-well plates at a density of 2 × 10^4^ cells/ml for 24 h. Cells were starved overnight and then pretreated with different concentrations of hyperoside for 24 h followed by incubation with LPS (1 *μ*g/ml) for another 24 h. The medium was refreshed and incubated with 50 *μ*l of MTT (5 mg/ml prepared in phosphate-buffered saline) for 4 h at 37°C. Next, the solution was removed and added DMSO to plates. The absorbance at 570 nm was measured using a plate reader.

### 2.4. Western Blotting

RIPA buffer added to HT22 cells (Invitrogen; USA) to collect the total protein; then the protein concentrations were determined assessed using the BCA method (Invitrogen, USA). The protein samples were mixed with a 5x loading buffer and denatured at the boil. The proteins were separated by 15% SDS-PAGE and transferred onto polyvinylidene fluoride membranes. Next, the membranes were incubated with primary antibodies (Bcl-2, Bax, caspase-3, BDNF, NGF, SIRT1, Wnt1, *β*-catenin, Shh, Patch, and GAPDH) at 4°C overnight. Then, the next day, the membranes were washed with PBS and incubated with secondary antibodies for 1 h. Finally, the protein bands were measured using the ImageJ software. The data were collected from at least three independent experiments.

### 2.5. qRT-PCR

Total RNA of HT22 cells was isolated using the TRIzol RNA Extraction Kit (Invitrogen, Grand Island, NY, USA), and the isolated total RNA was reverse-transcribed to cDNA using a reverse transcription kit (Takara, Kyoto, Japan). Next, SYBR Premix Ex Taq II (Takara, Kyoto, Japan) was used to perform qRT-PCR amplification. The IL-1*β*, IL-6, and TNF-*α* amplification primers were as follows: IL-1*β*, 5′-GATGGTCGCATTAGCTCC-3′ and 5′-GGCTGTAGCTGTAGCGTC-3′; IL-6, 5′-ATTGCGGCGGCTGACGCGTAG-3′ and 5′-GTCTGTTGCGCGAGCTGGTA-3′; IL-8, 5′-GTCGAGCTGCCGCGTAGCGT-3′ and 5′-CGCGATGCGTGCAGC-3′; and TNF-*α*, 5′-CGTCAGCCGATTTGCTATCT-3′ and 5′-CGGACTCCGCAAAGTCTAAG-3′. The relative expression of mRNA was analyzed using the 2^-*ΔΔ*Ct^ method.

### 2.6. SOD, GSH, and MDA Assay

We measured oxygen species (ROS), catalase (CAT), superoxide dismutase (SOD), glutathione (GSH), and malondialdehyde (MDA) levels activity using the corresponding assay kits (Nanjing Jiancheng Bio Company, China).

### 2.7. Flow Cytometry

Flow cytometry assay was used to measure the cell apoptosis rate according to a previous report [15]. HT22 cells in each group were harvested and resuspended. The apoptotic cells were double-labeled with annexin V-FITC and PI using an annexin V-FITC/PI apoptosis detection kit (Beyotime Biotechnology, China) for 30 min at room temperature in the dark. Then, the fluorescence intensity of the cells was quantified by flow cytometry.

### 2.8. Statistical Analysis

In this study, the difference between two groups was compared by using a *t*-test, and that among groups was analyzed by one-way analysis of variance (ANOVA). All data were presented as the mean values ± standard deviation (SD). Statistical analyses were performed by GraphPad Prism 7.0 software. *P* < 0.05 was considered to indicate a statistically significant difference.

## 3. Results

### 3.1. Hyperoside Alleviated Apoptosis and Inflammation in the LPS-Induced HT22 Cells

**Figure 1:**
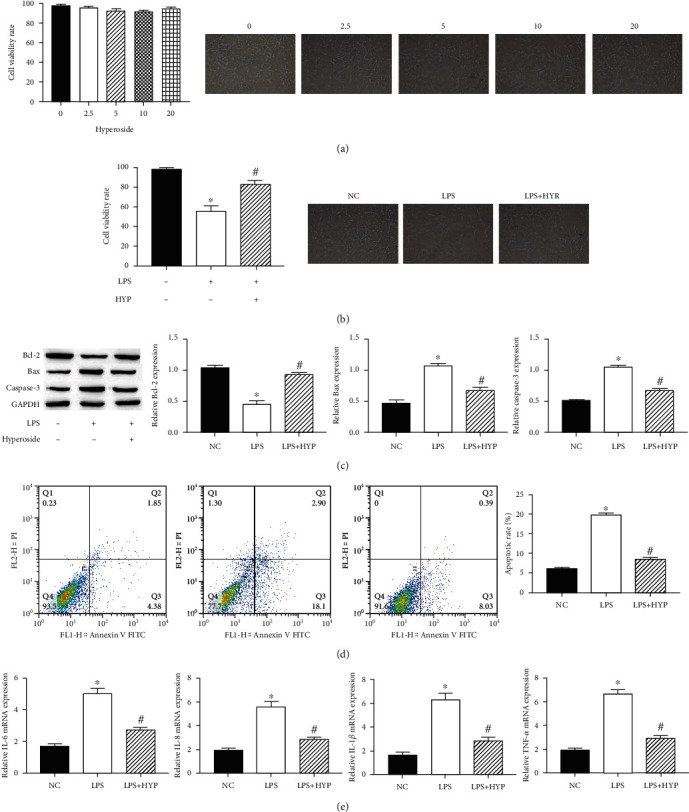
Hyperoside alleviated apoptosis and inflammation in the LPS-induced HT22 cells. HT22 cell viability was measured by the MTT assay. Cell viability was observed by a microscope (100x) (a, b). The expression of Bcl-2, Bax, and caspase-3 in HT22 cells was measured by western blotting (c). The HT22 cell apoptosis rate was measured by flow cytometry assay (d). The levels of IL-1*β*, IL-6, IL-8, and TNF-*α* mRNA in HT22 cells were measured by qRT-PCR (e); ∗ was considered significant compared to control (^∗^*P* < 0.05); # was considered significant compared to LPS (^#^*P* < 0.05).

The viability of HT22 cells was detected using the MTT assay. The result showed that hyperoside had little effect on HT22 cell viability (Figure 1(a)). Compared with untreated cell, the treatment of LPS (1 *μ*g/ml) significantly reduced HT22 cell viability (Figure 1(b)). Based on the MTT results, we choose 20 *μ*M hyperoside to evaluate the protective effect on HT22 cells. The apoptotic protein expression of HT22 cells was detected using the western blotting; the results showed that treatment with 20 *μ*M hyperoside significantly enhanced the antiapoptotic Bcl-2 expression and decreased the proapoptotic Bax and caspase-3 expression (Figure 1(c)). Simultaneously, 20 *μ*M hyperoside treatment significantly inhibited LPS-induced apoptosis of HT22 cells (Figure 1(d)). Likewise, treatment with 20 *μ*M hyperoside suppressed LPS-induced production of IL-1*β*, IL-6, IL-8, and TNF-*α* (Figure 1(e)). These results suggested that hyperoside alleviated apoptosis and inflammation in the LPS-induced HT22 cells.

### 3.2. Hyperoside Alleviated Oxidative Stress and Reduction of Neurotrophic Factor in the LPS-Induced HT22 Cells

**Figure 2:**
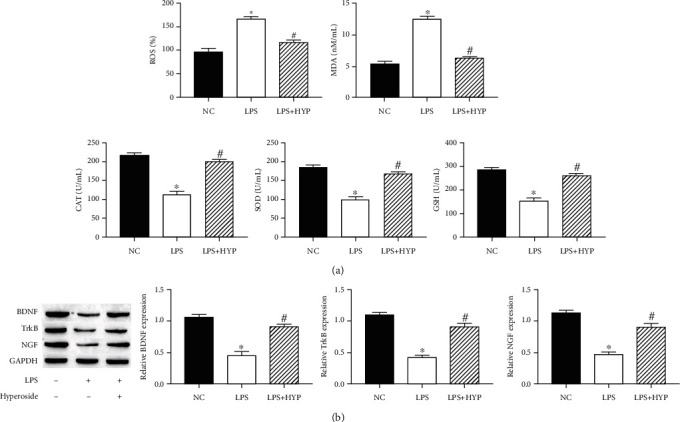
Hyperoside alleviated oxidative stress and reduction of neurotrophic factor in the LPS-induced HT22 cells. The levels of SOD, GSH, CAT, ROS, and MDA were measured by corresponding assay kits (a). The expression of BDNF, TrkB, and NGF in HT22 cells was measured by western blotting (b). ∗ was considered significant compared to control (^∗^*P* < 0.05); # was considered significant compared to LPS (^#^*P* < 0.05).

The oxidative stress of HT22 cells increased, and the production of neurotrophic factors decreased after LPS treatment. Next, we studied the effect of hyperoside on oxidative stress and neurotrophic factors in LPS-induced HT22 cells. The results showed that treatment with 20 *μ*M hyperoside significantly increased the levels of SOD, GSH, CAT, and neurotrophic factors BDNF, TrkB, NGF and decreased the levels of ROS and MDA (Figures 2(a) and 2(b)). These results suggested that hyperoside alleviated LPS-induced oxidative stress and reduction of neurotrophic factor in HT22 cells.

### 3.3. Hyperoside Inhibits LPS-Induced HT22 Cell Apoptosis and Inflammation through SIRT1

**Figure 3:**
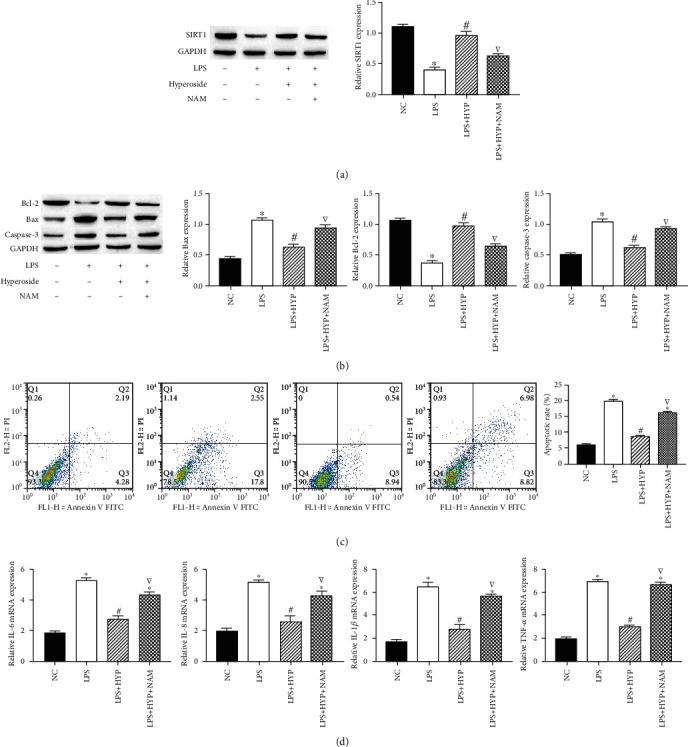
Hyperoside inhibits LPS-induced HT22 cell apoptosis and inflammation through SIRT1. The expression of SIRT cells was measured by western blotting (a). The expression of Bcl-2, Bax, and caspase-3 in HT22 cells was measured by western blotting (b). The HT22 cell apoptosis rate was measured by flow cytometry assay (c). The level of IL-1*β*, IL-6, IL-8, and TNF-*α* mRNA in HT22 cells was measured by qRT-PCR (d). ∗ was considered significant compared to control (^∗^*P* < 0.05); # was considered significant compared to LPS (^#^*P* < 0.05); ▽ was considered significant compared to LPS+HYP (^▽^*P* < 0.05).

SIRT1 alleviated nerve damage; to find out whether hyperoside could ameliorate apoptosis and inflammation in the LPS-induced by acting on SIRT1, SITR1 inhibitors (nicotinamide (NAM)) were used to treat cells. Western blotting showed that SITR1 expression decreased in LPS-induced cells; simultaneously, compared with LPS induction, hyperoside treatment upregulated the expression level of SITR1. In addition, NAM treatment decreased the expression of SIRT1 in HT22 cells compared with the treatment of cells with LPS+hyperoside treatment (Figure 3(a)). Likewise, NAM treatment decreased the expression of Bcl-2 and enhanced the expression of Bax and caspase-3 in HT22 cells compared with the treatment of cells with LPS+hyperoside treatment (Figure 3(b)). Simultaneously, NAM treatment significantly increased apoptosis of HT22 cells (Figure 3(c)). In addition, NAM treatment increased the expression of IL-1*β*, IL-6, IL-8, and TNF-*α*, compared with the treatment of cells with LPS+hyperoside treatment (Figure 3(d)). These results indicated that hyperoside inhibits LPS-induced HT22 cell apoptosis and inflammation by upregulating SIRT1.

### 3.4. Hyperoside Inhibits LPS-Induced HT22 Cell Oxidative Stress and Reduction of Neurotrophic Factor through SIRT1

**Figure 4:**
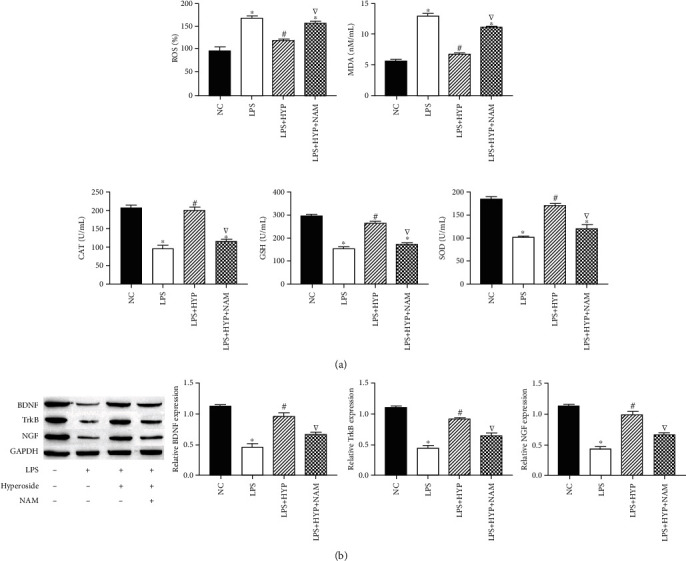
Hyperoside inhibits LPS-induced HT22 cell oxidative stress and reduction of neurotrophic factor through SIRT1. The level of SOD, GSH, CAT, ROS, and MDA were measured by corresponding assay kits (a). The expression of BDNF, TrkB, and NGF in HT22 cells was measured by western blotting (b). ∗ was considered significant compared to control (^∗^*P* < 0.05); # was considered significant compared to LPS (^#^*P* < 0.05); ▽ was considered significant compared to LPS + HYP (^▽^*P* < 0.05).

We studied the effect of SIRT1 on oxidative stress and neurotrophic factors in LPS-induced HT22 cells. The results showed that NAM treatment decreased the levels of SOD, GSH, CAT, and neurotrophic factors BDNF and NGF and increased the levels of ROS and MDA in HT22 cells, compared with the treatment of cells with LPS+hyperoside treatment (Figures 4(a) and 4(b)). These results indicated that hyperoside inhibits oxidative stress and neurotrophic factor reduction in LPS-induced HT22 cells by upregulating SIRT.

### 3.5. Hyperoside Activates Wnt/*β*-Catenin and Sonic Hedgehog Pathways by Upregulating SIRT1

**Figure 5:**
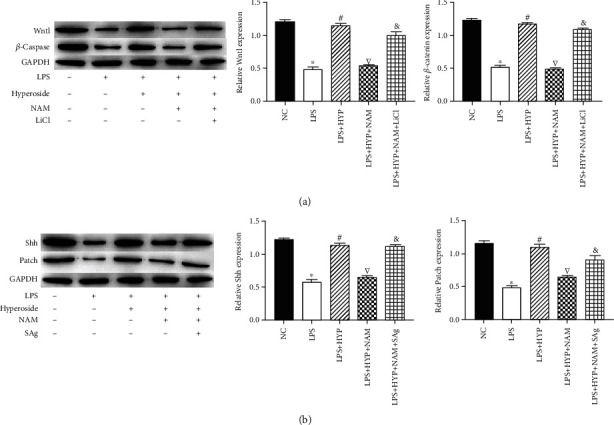
Hyperoside activates Wnt/*β*-catenin and sonic hedgehog pathways by upregulating SIRT1. The expression of Wnt1 and *β*-catenin in HT22 cells was measured by western blotting (a). The expression of Shh and patch in HT22 cells was measured by western blotting (b). ∗ was considered significant compared to control (^∗^*P* < 0.05); # was considered significant compared to LPS (^#^*P* < 0.05); ▽ was considered significant compared to LPS+HYP (^▽^*P* < 0.05); & was considered significant compared to LPS+HYP+LiCl or LPS+HYP+SAg (^&^*P* < 0.05).

To clarify whether SIRT1 alleviates HT22 damage through the Wnt/*β*-catenin and sonic hedgehog pathways, we measured the expression levels of signaling molecules in the Wnt/*β*-catenin and sonic hedgehog pathways. The results showed that LPS significantly decreased the expression levels of Wnt1, *β*-catenin, Shh, and Patch; hyperoside treatment significantly increased the expression levels of Wnt1, *β*-catenin, Shh, and patch; in addition, NAM treatment decreased the expression levels of Wnt1, *β*-catenin, Shh, and patch. In addition, in HT22 cells, compared with NAM-treated cells, Wnt/*β*-catenin agonist (LiCl) and sonic hedgehog agonist (SAg) inhibited the inhibitory effect of NAM (Figures 5(a) and 5(b)). These results indicated that hyperoside activates Wnt/*β*-catenin and sonic hedgehog pathways by upregulating SIRT1.

## 4. Discussion

Neuroinflammation is associated with the pathology of many neurological complications, including hearing loss, AD, PD, neuropathic pain, cognitive impairment, and cerebral ischemic injury [2, 16–24]. Identification of effective inflammatory protective candidate agents is one of the hot spots in the treatment of neurological complications [25]. Effective medical treatment reduces neuroinflammation or prevents neurodegeneration. Hyperoside has been reported to treat neuroinflammation in neurological complications [12], However, in recent years, there has been little research on the mechanism by which hyperoside alleviates neuroinflammation. In the present study, we explored the anti-inflammatory effects of hyperoside on LPS-induced HT22 neuroinflammation in mouse neuronal cells. We demonstrated that hyperoside attenuated apoptosis, inflammation, and oxidative stress and simultaneously restored the levels of neurotrophic factor proteins BDNF, TrkB, and NGF in HT22 cells induced by LPS. In addition, we provide evidence that STRI1 is highly expressed under the action of hyperoside and activates Wnt/*β*-catenin and sonic hedgehog pathways. In our study, hyperoside significantly inhibited the LPS-induced apoptosis, inflammation, and oxidative stress production by increased STRI and activating the Wnt/*β*-catenin and sonic hedgehog pathways.

Neurons are the basic structural and functional units of the nervous system [26]. Changes in the structure and function of neurons in the brain will cause nerve damage [27]. HT22 cells are a kind of mouse hippocampal neuronal cells, which are widely used as an in vitro neuronal model associated with neuroinflammation and nerve injury in studies to identify effective inflammatory protective candidate agents [28]. Previous studies have shown that systemic administration of LPS triggers nerve injury and neuroinflammation and in the brain, which induce neurodegeneration in mice [29, 30]. In the present study, we used HT22 cells as a neuronal cell model to examine the protective effect of hyperoside on LPS-activated neuroinflammation. We found that LPS inhibited HT22 activity, promoted the level of proinflammatory cytokines (TNF-*α*, IL-1*β*, IL-6, and IL-8) and apoptosis proteins Bax and xaspase-3, and activated oxidative stress. However, pretreatment with hyperoside significantly protected HT22 cells from LPS-induced cell growth inhibition by inhibiting apoptosis; downregulating TNF-*α*, IL-1*β*, IL-6, IL-8, Bax, and caspase-3 levels; and inhibiting oxidative stress. These results are consistent with previous studies on hyperoside as antioxidants, anti-inflammatory agents, and antiapoptotic agents.

Regulation that protects neuronal survival is essential in the pathological process of alleviating neurological complications caused by neuroinflammation [2, 31]. In the development of neuronal, BDNF, as an important nerve growth factors, plays an important role in the growth, survival, and differentiation of neurons [32]. BDNF enhances neuronal survival and protects synaptic function by binding to tropomyosin receptor kinase B (TrkB) [33, 34]. Simultaneously, BDNF promotes neuronal outward growth and recombination of dendritic spines, thereby improving neuronal connectivity [35]. NGF is a nerve cell growth regulator with dual biological functions of neuron nutrition and neurite outgrowth promotion. It has been proven to protect neurons by promoting nerve fiber regeneration [36, 37]. Previous studies have shown that LPS-induced inflammation is the main cause of neuronal death in the hippocampus. In this study, we found that the expression levels of BDNF, TrkB, and NGF in HT22 cells induced by LPS were significantly reduced and hyperoside treatment significantly restores the expression levels of BDNF, TrkB, and NGF.

SIRT1 is the main regulator of neurogenesis and plays a neuroprotective role in neurological diseases [38]. Previous studies showed that activation of SIRT1 reversed nerve damage in different neurological diseases by augmenting hippocampal neurogenesis [39]. Simultaneously, previous reports also demonstrated that activation of SIRT1 reduces the level of oxidative stress and the extent of inflammation [40]. In addition, crucially, Li et al. found that hyperoside enhances SIRT1 protein expression in a mechanism that protects ECV-304 cells from tert-butyl hydrogen peroxide-induced damage [41]. Thus, we speculate that hyperoside alleviating neuroinflammation may be related to the level of SIRT1. In this study, we found that hyperoside significantly increased the expression of SIRT1 in cells, while the SIRT1 inhibitor NAM effect attenuated the alleviating effect of hyperoside on neuroinflammation of induced by LPS. Wnt/*β*-catenin and sonic hedgehog pathways are confirmed to be regulated by SIRT1; SIRT1-mediated deacetylation in the process of c-myc degradation, which affected the stability of c-myc and increased the transcriptional activity of *β*-catenin, activates Wnt signaling through *β*-catenin [42]; simultaneously, SIRT1 agonist SRT1720 activated the sonic hedgehog signaling [43]. In addition, we have also observed that Wnt/*β*-catenin and sonic hedgehog signaling pathways are inhibited in neuroinflammation [44, 45]. Simultaneously, Wnt/*β*-catenin and sonic hedgehog signaling pathways are involved in the development of neuroinflammation-mediated hearing loss and other neurological diseases [46–55]. In this study, we found that hyperoside activates the expression of Wnt1, *β*-catenin, Shh, and patch by upregulating SIRT1.

In summary, the present study showed that hyperoside alleviated apoptosis, inflammation, oxidative stress, and reduction of neurotrophic factor in the LPS-induced HT22 cells. We further found that hyperoside alleviated nerve damage by upregulating SIRT1 to activate Wnt/*β*-catenin and sonic hedgehog signaling pathways. In conclusion, based on our findings, the therapeutic effect of hyperoside on neuroinflammation is further clarified, providing possible treatment basis for its clinical application for neuroinflammation.

## Data Availability

The data used to support the findings of this study are available from the corresponding author upon reasonable request.
